# Impact of time-restricted eating on reproductive hormones and androgen markers in adult females: A systematic review

**DOI:** 10.1177/02601060261467643

**Published:** 2026-07-10

**Authors:** Aimee Jirapak, Emily Adams, Georgia M Black, Brooke L Devlin

**Affiliations:** 1 Faculty of Health, Medicine and Behavioural Sciences, School of Human Movement and Nutrition Sciences, The University of Queensland, Brisbane, QLD, Australia

**Keywords:** Time-restricted eating, intermittent fasting, female reproductive hormones, androgens, polycystic ovary syndrome (PCOS), systematic review, menstrual cycle, sex hormones

## Abstract

**Background:**

Time-restricted eating (TRE) is a popular dietary intervention shown to facilitate weight loss. However, the impact of TRE on female reproductive hormones and androgen markers is not clear.

**Aim:**

This systematic review aimed to synthesise and evaluate the current body of evidence on the effect of TRE on reproductive hormones and androgen markers in adult females.

**Methods:**

A systematic search was conducted across PubMed, Embase, CINAHL and Web of Science. All randomised controlled trials, pre–post and pilot/feasibility studies until May 2024 meeting the predefined inclusion criteria were included. Two researchers screened and selected eligible studies and extracted relevant data. Eligible studies methodological quality was assessed using the American Dietetic Association (ADA) Quality Criteria Checklist.

**Results:**

Six studies met the inclusion criteria. Randomised controlled trials (RCTs) conducted in women with general obesity (body mass index > 30 kg/m^2^) consistently found no significant changes in androgens or other key hormones (testosterone, oestrogen, progesterone, luteinising hormone, cortisol, dehydroepiandrosterone and sex hormone-binding globulin). In contrast, non-randomised studies focusing on women with polycystic ovary syndrome (PCOS) reported beneficial changes to hormone profiles, including decreased testosterone and luteinising hormone, although these effects may have been confounded by weight loss.

**Conclusion:**

While limited by six studies, the current evidence on TRE's impact on female hormones is inconclusive and varies significantly by population and study design. While TRE appears to be a hormonally safe weight-loss strategy in women with general obesity, more rigorous, long-term RCTs are needed to establish its therapeutic role, in particular for conditions like PCOS.

## Introduction

Time-restricted eating (TRE) is a dietary approach that limits energy intake to a specific window of time within a 24-hour period, usually between 4 and 14 h, following by fasting in the subsequent hours (Ezpeleta et al., 2024; Park et al., 2021). The time of TRE protocols vary with a common regime being 16-hour fast and 8 hour eating window (16:8) and can vary with the eating window beginning early in the day (early TRE; 7 a.m.–3 p.m.) to late in the day (late TRE; 12 noon–8 p.m.) (Parr et al., 2022). This temporal dietary pattern is designed to align with the body's circadian rhythm, a natural, internal process that regulates sleep, metabolism and hormone secretion across a 24-hour cycle (BaHammam and Pirzada, 2023). Disruptions to this circadian synchronisation, such as food consumption during atypical hours, can increase susceptibility to metabolic disorders (BaHammam and Pirzada, 2023). In recent years, TRE has gained popularity due to its simplicity as it focuses on adjusting the timing of food and fluid consumption rather than requiring the elimination of specific foods, or the restriction of energy and macronutrients (Parr et al., 2022).

Literature indicates that TRE offers an array of health benefits across diverse populations (Chang et al., 2024). Specifically, TRE has been shown to reduce body weight and adiposity (Kalam et al., 2022; Kotarsky et al., 2021; Lin et al., 2024) which is likely to mediate improvements in several cardiometabolic outcomes (Kalam et al., 2022; Kortarsky et al., 2021; Lin et al., 2024). In addition to these weight-loss associated benefits, there is evidence that TRE facilitates weight-independent metabolic improvements. For example, early TRE improved insulin sensitivity and fasting glucose without significant weight change (Sutton et al., 2018). Blood pressure improvements have been observed with TRE, though these appear more consistently in parallel with weight reduction (Patterson and Sears, 2017). These positive health outcomes have been observed in individuals with obesity (Ezpeleta et al., 2024), prediabetes, type 2 diabetes (Cienfuegos et al., 2021), and polycystic ovarian syndrome (PCOS) (Feyzioglu et al., 2023; Li et al., 2021; Talebi et al., 2024), as well as across varying life stages, such as menopause (Kalam et al., 2022). While the metabolic benefits of TRE have been documented, a significant gap remains in understanding how this dietary pattern directly influences female sex hormones.

When following TRE, the deliberate alteration of food timing triggers a metabolic shift at both a cellular and systemic level (Longo and Panda, 2016). During the fasting periods inherent to TRE, a decrease in energy availability signals to the hypothalamus to reduce stimulation of the hypothalamic-pituitary-gonadal (HPG) axis (Acevedo-Rodriguez et al., 2018). The HPG axis is the primary regulator of reproductive hormones, controlling the production of luteinising hormone (LH), oestrogen, progesterone and testosterone (Acevedo-Rodriguez et al., 2018). When signalling pathways within the HPG axis are suppressed, gonadotropin-releasing hormone release is reduced, which can lead to lower levels of LH and interfere with the normal hormonal cascade required for healthy ovulation, regular menstrual cycles and overall female health (Acevedo-Rodriguez et al., 2018; Li et al., 2021). Furthermore, the physiological stress induced by fasting can cause cortisol levels to rise, which can further suppress the HPG axis and disrupt oestrogen, progesterone and testosterone production (Acevedo-Rodriguez et al., 2018; Chawla et al., 2021). These mechanisms highlight the potential for TRE to adversely affect menstrual function, particularly if fasting duration exceed an individual's energy availability threshold. In contrast, women with PCOS exhibit a fundamentally different endocrine profile which may alter their physiological response to TRE (Mao et al., 2024). There is a critical need to understand how fasting during TRE may interact with the sensitive hormonal regulation in females with and without PCOS.

To date, there have been no comprehensive reviews focused specifically on the impact of TRE on female reproductive and androgen markers. A thorough understanding of this relationship is essential for the advancement of women's healthcare, allowing both women and their healthcare providers to make informed, evidence-based decisions (Acevedo-Rodriguez et al., 2018; Draper et al., 2018). This systematic review aims to synthesise existing research to better understand the effect of TRE on hormonal levels (including testosterone, oestrogen, progesterone, LH, cortisol, dehydroepiandrosterone (DHEA) and sex hormone-binding globulin (SHBG)) in women, identify patterns across different populations and study designs, and ultimately, lay the foundation for more targeted health interventions tailored to the female population.

## Methods

### Study design

This review was conducted in accordance with 2020 Preferred Reporting Items for Systematic Reviews and Meta-Analyses (PRISMA) guidelines (Page et al., 2021). The protocol was registered with Prospero (CRD420251138025).

### Eligibility criteria

The Population, Intervention, Comparator, Outcome and Study Design (PICOS) principle guided the eligibility criteria for this study (Table 1). Studies were included if they met the following inclusion criteria: (1) adult female participants (>18 years), with or without a chronic disease; (2) use of TRE as the primary intervention, regardless of the TRE protocol; (3) assessment of changes to oestrogen, progesterone, cortisol, LH, testosterone, DHEA and sex-hormone-binding globulin levels; (4) published in English; and (5) all originally published RCTs, pre–post trials, prospective cohort and pilot/feasibility studies. Studies were therefore excluded if they met the following exclusion criteria: (1) non-English language; (2) non-peer reviewed articles or opinion pieces; (3) animal studies; (4) participants younger than 18 years old; (5) male data; (6) religious fasting practices and (7) intervention durations of less than a week.

**Table 1.** Population, Intervention, Comparator, Outcome and Study Design (PICOS) criteria for inclusion of studies.

Parameter	Criteria
Population	Adult females aged >18 years, with or without chronic disease
Intervention	Time-restricted eating as the primary intervention
Control	Inclusion/exclusion of a control or comparator group
Outcomes	Hormonal/androgen markers including luteinising hormone, all types of oestrogen (oestradiol, oestrone and oestrogen), progesterone, testosterone, and cortisol, dehydroepiandrosterone and sex hormone-binding globulin.
Study design	All originally published randomised controlled trials, pre–post trials, prospective cohort and pilot/feasibility studies

### Search strategy

The search criteria were developed to ensure a comprehensive retrieval of relevant literature across the specified databases. The following search criteria were applied: TITLE-ABS-KEY (“Women” OR Female OR women OR sex OR gender) AND (“Intermittent Fasting” OR TRE OR “time-restricted eating” OR “intermittent fast*”) AND (“Hormones” OR Hormone* OR Androgen* OR Oestrogen* OR Progesterone OR Cortisol). Search strings were manually translated for each database, and an electronic search was conducted across PubMed, CINAHL, Web of Science and Embase. No restrictions on publication dates were applied. All published were collated using EndNote X9 software (Clarivate, Philadelphia, PA), where duplicates were identified and removed.

### Selection process

All publications were imported into Covidence software to prepare for screening. Authors (EA and BD) independently screened the records by title and abstract of identified studies and assessed them against the inclusion criteria. This was followed by a full-text review of the remaining studies by the same two researchers to determine the research used in data extraction. No conflicts arose and thus a third party was not required. After the selection process, all eligible articles were forward-searched and their reference lists screened to identify any studies missing in the initial literature search. All papers published before May 2025 that met the eligibility criteria were included.

### Data collection process

Data was extracted from all eligible studies by two authors (EA and AJ) using an adapted template in Microsoft Word (Microsoft Corporation, Redmond, WA, USA). This information was organised into two tables detailing study characteristics and quantitative outcomes. Extracted data points included: subject population (age and health status), sample size, study design, intervention type and eating window, study duration, hormones and androgens investigated, the main objectives of the study, and the key outcomes such as weight loss and quantitative hormonal changes (including baseline and end-of-study values, absolute changes and statistical significance).

### Methodological quality assessment

The methodological quality and risk of bias of each included study were conducted using the American Dietetic Association (ADA) Quality Criteria Checklist for Primary Research. This tool is widely used in nutrition research reviews and aligns with the methodological diversity of included studies. This checklist evaluates each study based on four relevance questions and ten validity questions; the validity questions assess potential bias in study design, subject selection, intervention description, outcome measurement, and statistical analysis. Based on the performance on the validity questions, each study was assigned an overall rating of positive (+), neutral (Ø) or negative (−). Each quality assessment was cross-checked by two researchers, and any conflicts were resolved.

### Data synthesis

A synthesis of the extracted data was performed to summarise the findings from the included studies. No meta-analysis was conducted. The results were grouped by the primary hormonal outcomes (e.g. testosterone and androgen markers, oestrogens and LH). The impact of TRE on these hormones was summarised, with differences in study design, methodological quality and population characteristics used to understand the findings. A meta-analysis was not conducted due to heterogeneity in study designs, populations and outcome measures.

## Results

### Literature search results

The results of the literature search are shown in Figure 1. An initial search across PubMed, Embase, CINAHL and Web of Science databases retrieved 787 studies, of which 492 references were removed using Covidence. A total of 295 articles proceeded to the abstract and title screening phase, which resulted in the exclusion of 256 studies. Of these, 37 articles were deemed eligible for full-text screening. Within this process, 31 articles were removed due to duplicates, abstracts, protocol papers, or ineligible outcomes, interventions, study designs and patient populations. Overall, six studies were identified as suitable for inclusion in this systematic review and were used for data extraction and analysis.

**Figure 1.**
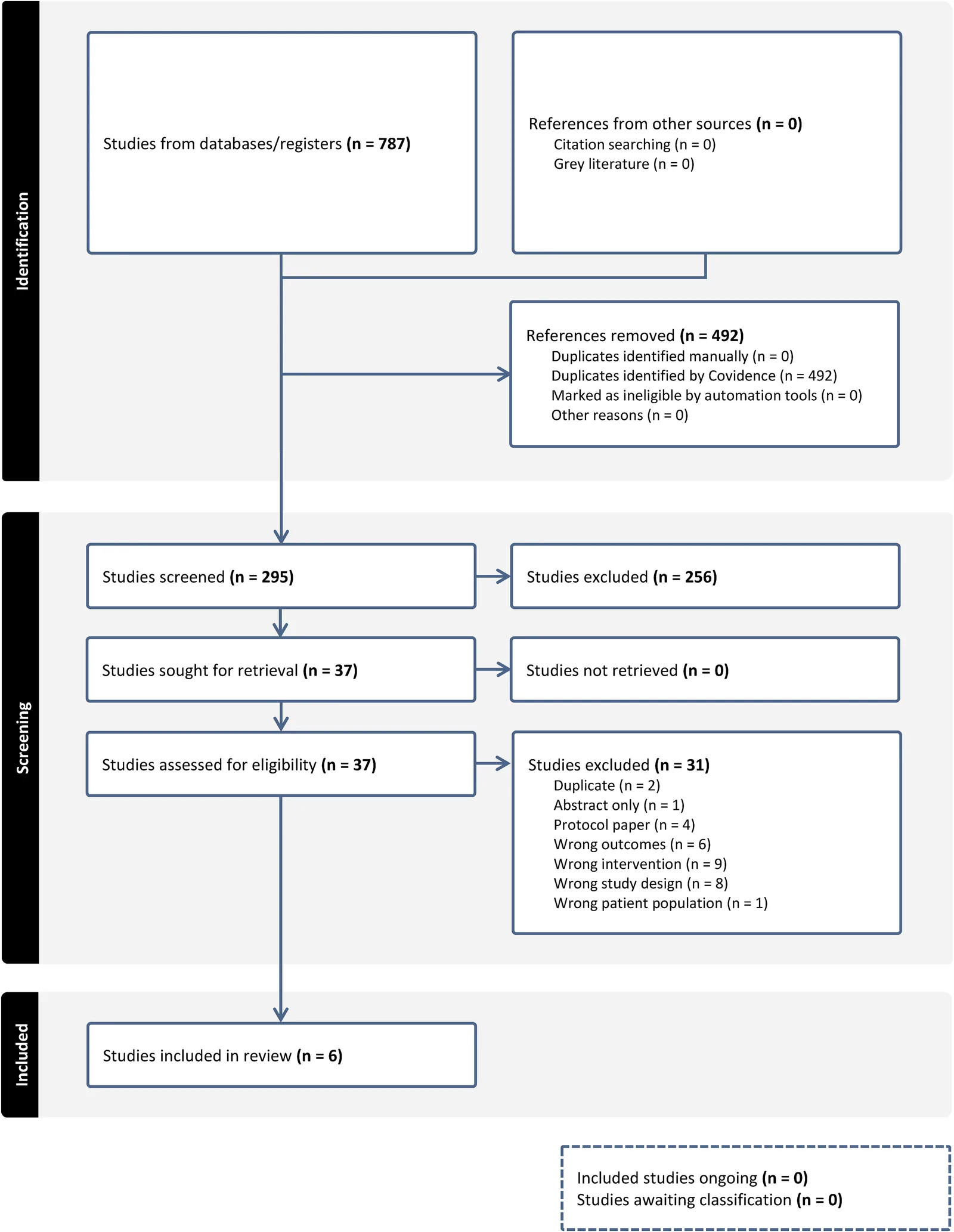
Preferred Reporting Items for Systematic Reviews and Meta-Analyses (PRISMA) flow diagram of results of literature search.

### Characteristics of included studies

The characteristics of the six included studies are outlined in Table 2. Across the studies, there was a total of 239 female participants, with study sample sizes ranging from *n* = 15 (Li et al., 2021) to *n* = 90 (Talebi et al., 2024). The studies were conducted across varying regions globally, including Iran (Talebi et al., 2024), Turkey (Feyzioglu et al., 2023), China (Li et al., 2021) and the USA (Kalam et al., 2022; Kotarsky et al., 2021; Lin et al., 2024). The study designs included two randomised controlled trials (RCTs) (Kotarsky et al., 2021; Talebi et al., 2024), two non-randomised pre–post trials (Feyzioglu et al., 2023; Li et al., 2021), and two secondary analyses of RCTs (Kalam et al., 2022; Lin et al., 2024).

**Table 2.** Characteristics of included studies.

Reference	Study design	Subject population	Sample size	Duration	Intervention	Hormone outcomes	Main study objective
Talebi et al. (2024)	8-week, 3-arm RCT	Overweight and obese women with PCOS via Rotterdam criteria (18–40 years, BMI 25–35 kg/m^2^)	90 (three groups of 30)	8 weeks	TRE: 14:10 (energy restricted; 8 a.m.–6 p.m. window) eTRE + probiotic versus eTRE + placebo versus DCR	Total testosterone, SHBG and DHEA-S (blood)	Compare eTRE (with/without probiotics) versus DCR on endocrine and metabolic profiles in women with PCOS
Feyzioglu et al. (2023)	6-week, retrospective pre–post study	Non-obese women with PCOS (18–40 years, mean BMI 25.1 kg/m^2^)	30	6 weeks	TRE: 16:8 (ad libitum; 1 p.m.–9 p.m. window)	Oestradiol, total/free testosterone and LH (blood)	Investigate effects of 8-h TRE on anthropometric, hormonal and metabolic profiles in women with PCOS
Li et al. (2021)	5-week, non-randomised pre–post trial	Overweight women with anovulatory PCOS (18–31 years, BMI ≥ 24 kg/m^2^)	15	5 weeks	TRE: 16:8 (ad libitum; 8 a.m.–4 p.m. window, set by researchers)	Total testosterone, LH, SHBG and FSH (blood)	To explore the effects of TRE on endocrine and metabolic profiles in women with anovulatory PCOS
Lin et al. (2024)	12-month RCT (secondary analysis)	Premenopausal (*n* = 44) and postmenopausal (*n* = 19) women with obesity (18–65 years, BMI 30–50 kg/m^2^)	73 (63 females)	12 months	TRE: 16:8 (energy restricted; 12 p.m.–8 p.m. window; widened to 10-h in maintenance) versus CR versus control	Total testosterone, DHEA, SHBG. Oestradiol, oestrone and progesterone (postmenopausal only) (blood)	TRE versus daily calorie restriction on sex hormone profiles females with obesity
Reference	Study design	Subject population	Sample size	Duration	Intervention	Hormone outcomes	Main study objective
Kotarsky et al. (2021)	8-week RCT	Overweight/obese adults (86% female, *n* = 18) (35–60 years, BMI 25–35 kg/m^2^)	21 (18 female)	8 weeks	TRE: 16:8 (ad libitum) + concurrent exercise versus normal eating + concurrent exercise (12 p.m.–8 p.m. window)	Oestradiol, progesterone, testosterone, DHEA-S, cortisol (saliva)	To determine if TRE combined with exercise reduces fat mass and preserves lean mass
Kalam et al. (2022)	8-week trial (secondary analysis of an RCT)	Premenopausal (*n* = 12) and postmenopausal (*n* = 11) women (18–65 years, BMI 30–49.9 kg/m^2^)	23	8 weeks	4-h and 6-h TRE eating windows (ad libitum; 1 p.m.–7 p.m. or 3 p.m.–7 p.m.)	Testosterone, androstenedione, DHEA, SHBG. oestradiol, oestrone and progesterone (postmenopausal only) (blood)	Effect of TRE on sex hormone levels in premenopausal and postmenopausal females

RCT: randomised controlled trial; PCOS: polycystic ovary syndrome; BMI: body mass index; DHEA: dehydroepiandrosterone; SHBG: sex hormone-binding globulin; TRE: time-restricted eating; eTRE: early time-restricted eating; DCR: daily calorie restriction.

Three studies explored the effects of TRE on women with polycystic ovary syndrome (PCOS) (Feyzioglu et al., 2023; Li et al., 2021; Talebi et al., 2024), while the remaining three investigated women who were overweight and obese and pre- and postmenopause (Kalam et al., 2022; Kotarsky et al., 2021; Lin et al., 2024). Across the studies, the age of participants ranged from 18 to 65 years. Specifically, the studies on women with PCOS covered age ranges between 18 and 40 years (Talebi et al., 2024; Feyzioglu et al., 2023; Li et al., 2021), while the studies in overweight and obese populations included women aged between 18 and 65 years (Kalam et al., 2022; Kotarsky et al., 2021; Lin et al., 2024). The included participants had a body mass index (BMI) ranging from 24 to 49.9 kg/m^2^ (Feyzioglu et al., 2023; Kalam et al., 2022; Kotarsky et al., 2021; Li et al., 2021; Lin et al., 2024). While two of the trials also recruited male participants, the data for those studies were analysed and presented separately for females (Kotarsky et al., 2021; Lin et al., 2024).

### TRE protocols

Three different TRE protocols were implemented in the studies included in this review with the duration of interventions ranging from 5 weeks (Li et al., 2021) to 12 months (Lin et al., 2024). The most prescribed eating window for TRE was the 16:8 protocol, although specific time windows varied between studies. Four studies utilised the 16:8 regimen, with interventions durations ranging from 5 weeks to 12 months (Feyzioglu et al., 2023; Kotarsky et al., 2021; Li et al., 2021; Lin et al., 2024). Other protocols included a 14:10 window (Talebi et al., 2024) and ad libitum 4-hour or 6-hour windows (Kalam et al., 2022). These protocols are outlined in Table 2.

### Hormonal and androgen marker outcomes

The primary hormonal and androgen marker outcomes from the six included studies are summarised in Table 3. Hormone levels were predominantly measured from fasting blood samples (Feyzioglu et al., 2023; Kalam et al., 2022; Talebi et al., 2024; Li et al., 2021; Lin et al., 2024) with one study measuring from saliva samples (Kotarsky et al., 2021). Only one study reported standardising sample collection to the early follicular phase of the menstrual cycle (Feyzioglu et al., 2023).

**Table 3.** Hormones/androgens changes of TRE intervention of included studies.

Reference	Population	Weight loss	Outcome measure	Baseline value (mean ± SD or median (IQR))	Post study value (mean ± SD ormedian (IQR))	Absolute change	*p*-value	Change
Studies on women with PCOS								
Talebi et al. (2024)	Overweight and obese women with PCOS (*n* = 90)	−2.9 kg (b)	Total Testosterone (ng/dL) (b)	40.5 ± 26.3	39.4 ± 16.6	−1.1	0.943	↔
			SHBG (nmol/L) (b)	11.1 ± 4.7	12.8 ± 6.1	+1.7	0.012	↑
			DHEA-S (µg/dL) (b)	178 ± 111	163 ± 79	−15	0.336	↔
Feyzioglu et al. (2023)	Non-obese women with PCOS (*n* = 30)	∼−3.0 kg (e)	Total Testosterone (ng/dL)	53.34 (25.48–77.15)	32.98 (13.18–41.57)	−20.36	<0.001	↓
			Free Testosterone (pg/mL)	1.75 (0.86–2.12)	0.78 (0.58–1.05)	−0.97	<0.001	↓
			Oestradiol (mIU/mL)	43.0 (35–52)	33.0 (30–39)	−10	<0.001	↓
			LH (mIU/mL)	9.95 (6.7–11.7)	5.23 (4.32–6.33)	−4.72	<0.001	↓
Li et al. (2021)	Overweight women with PCOS (*n* = 15)	−1.3 kg (−1.7%)	Total Testosterone (ng/mL)	1.00 (0.77–1.26)	0.91 (0.61–1.09)	−0.09	0.048	↓
			SHBG (nmol/L)	19.00 (11.70–25.10)	22.70 (15.20–33.90)	+3.70	<0.001	↑
			LH (mIU/mL)	13.09 ± 4.83	10.67 ± 5.22	−2.42	0.176	↔
			FSH (mIU/mL)					↔
Studies on women without PCOS								
Lin et al. (2024) (c)	Premenopausal women (*n* = 63); Postmenopausal women (*n* = 19)	3.7 kg (pre); 3.5 kg (post)	Total testosterone	Not reported	Not reported	No change	>0.05	↔
			SHBG	Not reported	Not reported	No change	>0.05	↔
			Oestradiol	Not reported	Not reported	No change	>0.05	↔
			Oestrone	Not reported	Not reported	No change	>0.05	↔
			Progesterone	Not reported	Not reported	No change	>0.05	↔
			DHEA-S	Not reported	Not reported	No change	>0.05	↔
Kotarsky et al. (2021)	Women with overweight/obesity (*n* = 18)	−3.0 kg (−3.3%	Testosterone (pg/mL)	27 ± 7	25 ± 7	−2	0.09	↔
			Oestradiol (pg/mL)	0.7 ± 0.1	0.9 ± 0.1	+0.2	0.228	↔
			Progesterone (pg/mL)	26 ± 12	27 ± 13	+1.0	0.181	↔
			DHEA-S (ng/mL)	4.7 ± 1.0	5.6 ± 1.4	+0.9	0.165	↔
			Cortisol (ng/mL)	5.4 ± 1.0	5.7 ± 0.6	+0.3	0.664	↔
Kalam et al. (2022)	All women (pre and post) (*n* = 23)	−3 to −4 kg (−3%)	DHEA (ng/mL)	1.58 ± 0.73	1.25 ± 0.61	−0.33	0.030 (d)	↓
			Total testosterone	Not reported	Not reported	No change	>0.05 (d)	↔
			Androstenedione	Not reported	Not reported	No change	>0.05 (d)	↔
			Oestradiol	Not reported	Not reported	No change	>0.05 (d)	↔
			Oestrone	Not reported	Not reported	No change	>0.05 (d)	↔
			Progesterone	Not reported	Not reported	No change	>0.05 (d)	↔
			SHBG	Not reported	Not reported	No change	>0.05 (d)	↔

↑: statistically significant increase; ↓: statistically significant decrease; ↔: no statistically significant change. SD: standard deviation; IQR: interquartile range; AMH: anti-Müllerian hormone; BMI: body mass index; CR: calorie restriction; DCR: daily calorie restriction; DHEA-S: dehydroepiandrosterone sulphate; eTRE: early time-restricted eating; FAI: free androgen index; FSH: follicle-stimulating hormone; LH: luteinising hormone; PCOS: polycystic ovary syndrome; RCT: randomised controlled trial; SHBG: sex hormone-binding globulin; TRE: time-restricted eating.

Footnotes: (b) Data shown for the eTRE + Placebo group to represent the TRE intervention without the probiotic confounder. The p-value reflects the within-group change from baseline, not the comparison to the DCR group.

(c) The Lin et al. paper reported no significant changes over time or between groups for any hormone but did not publish the specific baseline and end-of-study numerical values in its main text or figures.

(d) *P*-value shown is for the main effect of time, indicating change across both pre- and postmenopausal groups combined.

(e) Estimated from significant BMI reduction reported in the study.

### Testosterone and androgen markers

As the most frequently assessed marker, total testosterone was measured in all six studies. Four of these studies, all of which were RCTs or secondary analyses, reported no significant changes in total testosterone levels following the TRE intervention (Kalam et al., 2022; Kotarsky et al., 2021; Lin et al., 2024; Talebi et al., 2024). In contrast, two pre–post studies focusing exclusively on women with PCOS reported significant decreases in testosterone; one study documented a drop from 53.34 ng/dL to 32.98 ng/dL (*p* < 0.001) (Feyzioglu et al., 2023), while another also found a significant reduction (Li et al., 2021).

### Sex hormone-binding globulin

Four studies assessed SHBG (Kalam et al., 2022; Li et al., 2021; Lin et al., 2024; Talebi et al., 2024). Of these, three reported no significant changes in SHBG levels (Kalam et al., 2022; Lin et al., 2024; Talebi et al., 2024). In contrast, one pre–post study in women with PCOS reported a significant increase in SHBG (from 19.0 to 22.7 nmol/L, *p* < 0.001) (Li et al., 2021).

### Oestrogens and progesterone

Oestrogen (as oestradiol or oestrone) and progesterone were measured in four studies (Feyzioglu et al., 2023; Kalam et al., 2022; Kotarsky et al., 2021; Lin et al., 2024). The specific context for these measurements varies significantly. Two studies assessed these hormones exclusively in postmenopausal women (Kalam et al., 2022; Lin et al., 2024), one study analysed a mixed-age group of women without controlling for menstrual cycle phase (Kotarsky et al., 2021), and one study measured oestradiol in premenopausal women with PCOS during the early follicular phase (Feyzioglu et al., 2023). Across three of these studies, no significant changes in oestradiol or progesterone were observed following the TRE intervention (Kalam et al., 2022; Kotarsky et al., 2021; Lin et al., 2024). The exception was one pre–post study that reported a significant decrease in oestradiol (from 43 to 33 mIU/mL, *p* < 0.001) in women with PCOS (Feyzioglu et al., 2023).

### Luteinising hormone

LH was measured in two studies involving women with PCOS (Feyzioglu et al., 2023; Li et al., 2021). One pre–post study reported a significant decrease in LH levels (from 9.95 to 5.23 mIU/mL, *p* < 0.001) (Feyzioglu et al., 2023), while one RCT (Talebi et al., 2024) and one other pre–post trial (Li et al., 2021) found no significant change in LH with TRE.

### Dehydroepiandrosterone

DHEA or its sulphate (DHEA-S) was measured in four studies (Kalam et al., 2022; Kotarsky et al., 2021; Lin et al., 2024; Talebi et al., 2024). One secondary analysis investigation reported a significant decrease in DHEA levels in both pre- and postmenopausal women after 8 weeks of TRE (Kalam et al., 2022). In contrast, three RCTs found no significant changes in DHEA or DHEA-S levels after TRE (Kotarsky et al., 2021; Lin et al., 2024; Talebi et al., 2024).

### Cortisol

Cortisol was measured in one RCT and showed no significant change after 8 weeks of TRE combined with exercise (Kotarsky et al., 2021).

### Methodological quality of included studies

The results of the risk-of-bias assessment are summarised in Table 4. Of the six included studies, three were rated as having a positive (+) quality (Kotarsky et al., 2021; Lin et al., 2024; Talebi et al., 2024), indicating a low risk of bias and strong methodology. The remaining three studies were rated as neutral (Ø) (Feyzioglu et al., 2023; Kalam et al., 2022; Li et al., 2021), indicating that while methodologically sound in many areas, they contained weaknesses that increased the potential risk of bias. The positive-rated studies were all RCTs or secondary analyses of RCTs. The neutral-rated studies were primarily non-randomised pre–post designs or secondary analyses with a higher inherent risk of bias. No studies were rated as negative.

**Table 4.** Risk-of-bias results using the ADA quality checklist.

	Relevance questions (a)				Validity questions (b)										
Reference	Q1	Q2	Q3	Q4	Q1	Q2	Q3	Q4	Q5	Q6	Q7	Q8	Q9	Q10	Overall rating
Talebi et al. (2024)	Y	Y	Y	Y	Y	Y	Y	Y	Y	Y	Y	Y	Y	Y	+
Kotarsky et al. (2021)	Y	Y	Y	Y	Y	Y	Y	Y	N	Y	Y	Y	Y	Y	+
Lin et al. (correct paper)	Y	Y	Y	Y	Y	Y	Y	Y	N	Y	Y	Y	Y	Y	+
Kalam et al. (2022)	Y	Y	Y	Y	Y	N	Y	Y	N	Y	Y	Y	Y	Y	Ø
Feyzioglu et al. (2023)	Y	Y	Y	Y	Y	N	N/A	Y	N	Y	Y	Y	Y	Y	Ø
Li et al. (2021)	Y	Y	Y	Y	Y	N	N/A	Y	N	Y	Y	Y	Y	Y	Ø

ADA: American Dietetic Association; Y: Yes; N: No; N/A: not applicable; U: unclear; +: positive quality; Ø: neutral quality.

Footnotes: (a) The four Relevance Questions from the ADA Quality Criteria Checklist are (1) Would implementing the studied intervention or procedure (if found successful) result in improved outcomes for the patients/clients/population group? (NA for some Epi studies). (2) Did the authors study an outcome (dependent variable) or topic that the patients/clients/population group would care about? (3) Is the focus of the intervention or procedure (independent variable) or topic of study a common issue of concern to dietetics practice? (4) Is the intervention or procedure feasible? (NA for some epidemiological studies). (b) The 10 Validity Questions from the ADA Quality Criteria Checklist are (1) Was the research question clearly stated? (2) Was the selection of study subjects/patients free from bias? (3) Were study groups comparable? (4) Was method of handling withdrawals described? (5) Was blinding used to prevent introduction of bias? (6) Were intervention/therapeutic regimens/exposure factor or procedure and any comparison(s) described in detail? Were intervening factors described? (7) Were outcomes clearly defined and the measurements valid and reliable? (8) Was the statistical analysis appropriate for the study design and type of outcome indicators? (9) Are conclusions supported by results with biases and limitations taken into consideration? (10) Is bias due to study's funding or sponsorship unlikely?

## Discussion

This systematic review highlights the current inconclusive evidence regarding the impact of TRE on female hormones and androgen markers. With just six studies meeting the inclusion criteria, the review reflects a notably limited evidence base. The study populations and TRE protocols of all six trials varied. Studies with significant hormonal changes were predominantly reported in non-randomised studies focusing on women with PCOS. In contrast, RCTs conducted in more diverse populations of women with obesity generally showed hormonal stability. This suggests that the effects of TRE are not uniform and may be heavily influenced by study methodology, TRE protocol and the underlying health status of the participants. The impact of TRE on female hormones cannot be determined from the six studies included, and more research is needed to better understand this relationship.

The two studies that reported the most significant hormonal changes, including decreases in testosterone and LH, were non-randomised, pre–post studies in women with PCOS (Feyzioglu et al., 2023; Li et al., 2021). The lack of a concurrent control group in these trials makes it difficult to determine if the observed hormonal shifts were a direct result of the TRE protocol itself, or simply a consequence of the associated weight loss. It is well-established that weight loss, regardless of the dietary method, can independently improve hormonal parameters, especially in hyperandrogenic states (Feyzioglu et al., 2023; Li et al., 2021). For instance, the significant hormonal changes reported by Feyzioglu et al. (2023) in their 6-week study were accompanied a significant BMI reduction, while participants in the 5-week Li et al. (2021) study loss an average of 1.3 kg alongside hormonal improvements. However, TRE could be implemented as an effective and simple intervention to facilitate weight loss in women with PCOS.

Furthermore, the underlying health status of the participants appears to be a key determinant of their hormonal response to TRE. Three studies focused exclusively on women with PCOS and showed some beneficial hormonal or clinical outcomes (Talebi et al., 2024; Feyzioglu et al., 2023; Li et al., 2021). These included significant decreases in total testosterone (Feyzioglu et al., 2023; Li et al., 2021) and a reduction in LH (Feyzioglu et al., 2023). These findings align with the known pathophysiology of PCOS, which is often characterised by elevated androgens and LH due to disrupted metabolism and insulin resistance (Daescu et al., 2023). Specifically, insulin resistance in PCOS can directly stimulate the ovaries to produce excess androgens while also reducing the liver's production of SHBG, thereby increasing the amount of biologically active free testosterone (Floyd et al., 2022; Longo and Panda, 2016). The improvements seen in these studies suggest that in a state of hormonal dysregulation, the metabolic shifts induced by TRE may help normalise pathways related to hyperandrogenism (Purwar and Nagpure, 2022). However, across all studies on women with PCOS, hormonal outcomes were not adjusted for weight loss. Consequently, weight loss remains a plausible alternative explanation for the reported hormonal improvements in PCOS. Based on these preliminary findings, TRE is a potential intervention for managing the hormonal features of PCOS, possibly via facilitating weight loss, however, further well-controlled studies are needed.

These results stand in contrast to the findings in women without a primary PCOS diagnosis. The studies involving mixed pre- and postmenopausal women with obesity – all of which also reported weight loss of ∼3% to 4% (Kalam et al., 2022; Kotarsky et al., 2021; Lin et al., 2024) – generally reported hormonal stability, particularly for androgens and oestrogens (Kalam et al., 2022; Kotarsky et al., 2021; Lin et al., 2024). This may indicate that in women with a generally regulated endocrine system, TRE acts primarily as a weight-loss tool without causing significant, nor independent shifts in sex hormone levels.

The complexity of female physiology, particularly the cyclical nature of hormone fluctuations across the menstrual cycle and different life stages, presents a major challenge in this field of research (Draper et al., 2018). This has contributed to a notable lack of research prioritising the hormonal impact of TRE, reflecting a broader history of female health being an under-researched field. A persistent and significant challenge across the included literature is the difficulty in accurately assessing female hormones without standardised timing relative to the menstrual cycle. This was an explicitly stated limitation in one study (Kalam et al., 2022), and it is the primary reason why key reproductive hormones like oestradiol and progesterone were not measured in premenopausal women in the largest trial (Lin et al., 2024). The implication of this methodological hurdle is that for premenopausal women, the current literature can, at best, only offer preliminary insights into the effects of TRE on androgens. Therefore, robust conclusions about its effects on oestrogen and progesterone cannot be drawn (Draper et al., 2018; Thiyagarajan et al., 2024). Future TRE trials in eumenorrheic, premenopausal participants should standardise sampling to a biologically appropriate phase, verify ovulation, collect morning, fasting samples and report contraceptive use, anovulatory cycles and cycle length variability.

Furthermore, menopausal status is a major confounding variable. Menopause is characterised by a natural reduction in ovarian oestrogen and progesterone production, which drives significant changes in metabolism, insulin sensitivity and fat distribution (Motlani et al., 2023). Only one study directly compared pre- and postmenopausal groups (Kalam et al., 2022). In that secondary analysis, few differences in hormonal response to TRE were found, except for a significant decrease in DHEA in both groups. This finding, however, was not replicated in other RCTs (Kotarsky et al., 2021; Lin et al., 2024; Talebi et al., 2024), leaving the effect of TRE on DHEA unclear. Without clear differentiation between these life stages in all studies, it is difficult to determine whether observed hormonal changes are due to the TRE intervention or the natural physiological shifts associated with menopause (Magraith and Jang, 2023; Motlani et al., 2023). Distinguishing between these populations is crucial, considering that pre-menopausal women possess a cyclical hormonal environment that is highly sensitive to changes in energy availability, where TRE may potentially disrupt the HPG axis (Draper et al., 2018; Thiyagarajan et al., 2024). On the contrary, postmenopausal women undergo a profound shift from ovarian to adrenal-derived sex hormones, accompanied by a decline in oestrogen that fundamentally alters insulin sensitivity along with fat distribution (Magraith and Jang, 2023; Motlani et al., 2023). Consequently, pre- and postmenopausal women may display conflicting metabolic and endocrine responses to the fasting rhythms of TRE (Kalam et al., 2022). Without a stratified analysis, research will risk masking effects which are population-specific, therefore making it not possible to determine if TRE serves as a protective metabolic tool, or a potential endocrine disruptor across different female life stages (Draper et al., 2018).

Hormonal changes can be influenced by numerous external factors beyond diet, including stress, exercise and food choices, making it difficult to attribute alterations solely to TRE (Clark and Mach, 2016). Cortisol, a key stress hormone, provides a clear example. In the single study that measured cortisol, levels did not change significantly (Kotarsky et al., 2021). However, this finding is difficult to interpret because cortisol follows a well-established diurnal rhythm where it typically peaks shortly after waking and declines steadily across the day before reaching its lowest levels at night (Chawla et al., 2021). As a result, cortisol concentrations can vary two-to three-fold within hours, meaning that a single saliva sample provides only a snapshot of this dynamic pattern. This limitation is particularly important in the context of TRE, as meal timing itself can modify cortisol secretion patterns. Evidence shows that skipping breakfast has been associated with increased cortisol, while skipping dinner may decrease evening cortisol but elevate it the next morning (Acevedo-Rodriguez et al., 2018). Without multiple samples it is not possible to determine whether TRE influences the amplitude, timing, or slope of cortisol rhythm. Beyond the timing of collection, the analytical methodology used for hormone assessment significantly influences the validity of findings, particularly for oestrogen in older populations. In postmenopausal women, oestradiol levels are often extremely low and can frequently fall below detection limits of standard immunoassays (Magraith and Jang, 2023; Motlani et al., 2023). The studies included in this review (Kalam et al., 2022; Kotarsky et al., 2021; Lin et al., 2024) predominantly utilised standard blood or saliva assays without specifying the use of high-sensitivity mass spectrometry. This methodological limitation therefore may restrict the reliability of the reported stability in oestradiol and progesterone levels among postmenopausal women, as the tools used may lack the sensitivity to detect subtle hormonal changes. Furthermore, the inclusion of a mandatory, concurrent exercise protocol in one of the RCTs (Kotarsky et al., 2021) adds another layer of complexity, making it challenging to isolate the metabolic effects of TRE from those of structured physical activity. The studies included in this review did not account for these nuances, representing a significant gap in understanding how different TRE feeding windows might interact with the body's stress response.

To our knowledge, this is the first systematic review to synthesise the evidence on the specific impact of TRE on female reproductive hormones and androgen markers. A notable strength of this review is the inclusion of a formal risk-of-bias assessment using the ADA Quality Criteria Checklist, which allowed for a more critical and nuanced interpretation of the varied results.

However, the conclusions of this review are constrained by the quality and heterogeneity of the available primary studies. Only six studies met the inclusion criteria, and these differed markedly in key methodological features, with differing populations (PCOS vs. general obesity), TRE protocols (eating windows ranging from 4 to 10 h), and intervention durations (5 weeks to 12 months). These inconsistencies make direct comparisons difficult and substantially reduce the ability to determine whether observed hormonal changes are attributable to TRE itself or to differences in how TRE was implemented. For example, shorter eating windows may induce greater caloric restriction or metabolic stress than longer windows, while longer interventions (i.e. 12 months) may allow hormonal adaptations to emerge that shorter protocols cannot capture. Furthermore, many of the included studies had small sample sizes (Kalam et al., 2022; Li et al., 2021) and short durations, preventing any conclusions about the long-term hormonal effects of TRE. The shortest interventions of 5 to 6 weeks would fail to cover more than one or two full menstrual cycles for most women, making it difficult to establish a clear trend in hormonal changes (Harris et al., 2016; Li et al., 2021; Thiyagarajan et al., 2024). Finally, the inclusion of non-randomised trials (Feyzioglu et al., 2023; Li et al., 2021) increases susceptibility to confounding factors, particularly weight loss, which itself can influence reproductive hormones. Collectively, these factors limit the certainty and generalisability of the overall conclusions.

For clinical practice, these findings suggest that TRE can be considered a safe weight-loss strategy for the general population of females with obesity without significant concerns of disrupting major sex hormones like testosterone and oestrogen. For women with PCOS, TRE shows promise as a tool to help manage hyperandrogenism (via weight loss), but TRE interventions must be tailored based on an individual's hormonal profile, age, menopausal status and overall health goals (four).

For future research, it is clear there is a critical need for large, long-term, well-powered RCTs that are specifically designed to assess sex hormones as a primary outcome. These trials must: (1) directly compare TRE against standard-of-care diets in women with and without PCOS; (2) standardise hormone collection according to menstrual cycle phase; (3) directly compare the effects of different eating window timings (e.g. early vs. late TRE) to assess the influence of circadian alignment; and (4) include distinct analyses for pre- and postmenopausal women. Such research is vital to definitively understand the relationship between TRE and female hormonal health. Beyond the six studies identified in this review, a search of international trial registries indicates that large-scale RCTs investigating TRE and female hormones, specifically in PCOS are underway indicating a rapidly expanding research landscape. The anticipated results from these trials will be vital to address the current gaps in evidence regarding the long-term endocrine effects of TRE and its potential as a targeted therapeutic intervention, particularly in patients with PCOS.

## Conclusions

Overall, this systematic review of six studies found that the current evidence regarding the impact of TRE on female reproductive hormones and androgen markers is inconclusive and highly variable by population. A notable pattern emerged from this review: women with PCOS often experienced beneficial hormonal changes, particularly improvements in hyperandrogenism, whereas studies in women with obesity consistently reported stable hormones. It is important to note, however, that the findings in the PCOS population were primarily from non-randomised studies, where the effects were likely confounded by weight loss.

This variability underscores the complexity of female hormonal regulation and is compounded by significant heterogeneity across studies in terms of participant populations, intervention durations and research methodologies.

Ultimately, this review suggests that while TRE appears to be a hormonally safe weight-loss strategy for the general female population, its use as a targeted therapy for conditions like PCOS remains promising but is current still at a preliminary level. An individualised approach is therefore essential in clinical practice. With a limited evidence base as found in this review, there is a clear and urgent need for large-scale, long-term RCTs – which standardise hormone collection according to menstrual cycle phase – to establish definitive, evidence-based guidelines for the application of TRE in women's health.

## Data Availability

N/A
